# Insights into Public Perception Towards Poultry Welfare, Egg Labelling, and Willingness to Pay Among Young Adults in Ghana

**DOI:** 10.3390/ani16071120

**Published:** 2026-04-07

**Authors:** Daniel Baba Abiliba, Emmanuel Nyamekye, Emmanuel Dongbataazie Piiru, Jacob Achumboro Ayang, Richard Dogbatse, Prince Nana Takyi, Benjamin Obukowho Emikpe

**Affiliations:** 1 Animal Welfare League, Agbogba, Accra GE-215-6837, Ghana; abiliba@animalwelfareleague.org (D.B.A.); emmanuelnyamekye169@gmail.com (E.N.); dongbataazie@gmail.com (E.D.P.); ayangjacob1@gmail.com (J.A.A.); 2 Department of Pharmacology, Kwame Nkrumah University of Science and Technology, Kumasi AK-385-1973, Ghana; richarddogbatse12@gmail.com; 3 School of Veterinary Medicine, Kwame Nkrumah University of Science and Technology, Kumasi AK-731-0297, Ghana; princetakyi037@gmail.com

**Keywords:** farmed animal welfare, consumer perception, poultry farming, egg production system, Ghana

## Abstract

Animal welfare in farmed animals is increasingly being identified as an integral part of ethical meat production; yet in most developing nations, including Ghana, little attention is being paid to this area of interest. The demand for chicken meat and egg products in Ghana has also increased because of rapid urbanisation and development; hence, public perception of poultry welfare is paramount in policy formulation and development in Ghana. This study investigates public perception of poultry welfare in Ghana, particularly laying hen farming. The study used a cross-sectional study and surveyed 1275 respondents aged 17 and older in Accra, Kumasi, and Tamale by collecting data in-person, and the questionnaire was administered using tablets or mobile devices. The study found that 69.1% of respondents poorly perceived farmed animal welfare, while 30.9% positively perceived farmed animal welfare in Ghana. There was a significant difference in perception levels among respondents in Accra and Kumasi, and those in Tamale, where respondents in Tamale indicated a slightly positive perception compared to those in Accra and Kumasi. Furthermore, 53.7% of respondents supported state intervention in farmed animal welfare, while 52.0% showed reluctance to pay a premium price for cage-free and free-range egg production in Ghana.

## Simple Summary

Farm animal welfare is gaining increasing recognition globally, but remarkably, very little is known regarding the perceptions of farm welfare and welfare-related food labeling among consumers in Ghana. This study investigated public perceptions of poultry welfare, in particular laying hens, support for egg-labeling schemes, and willingness to pay premium prices for eggs produced under improved welfare conditions in three main cities in Ghana, namely Accra, Kumasi, and Tamale. Using a survey design, 1275 respondents among the adult population revealed a majority who lacked adequate perceptions and understanding of farm animal welfare, although many thought chickens should not suffer pain and supported the role of the government in promoting welfare standards. While most respondents supported the introduction of free-range and cage-free egg labeling schemes and perceived them as useful in promoting consumer decision-making, over half of them would not pay extra costs for eggs produced under improved welfare conditions. Pet ownership and ethnicity correlated with better welfare perceptions. This study reveals a gap between public welfare concerns and purchasing decisions, which appear largely driven by cost considerations.

## 1. Introduction

Farm animal welfare is increasingly at the forefront of global concerns. Ethical, welfare, environmental, and health concerns tied to farm animal agriculture and how animals are raised are driving the debate into action [1,2]. Meanwhile, Africa’s population is booming and projected to reach about 2.5 billion by 2050 [3]. With this demographic surge and a growing middle class, meat consumption, particularly poultry, is on the rise. In Ghana, economic growth and urbanisation are greatly shaping food choice, and poultry meat has become a household staple due to its lower cost and nutritional value [4].

The local poultry industry in Ghana is set for growth, with consumption of poultry meat projected to increase in the coming years, as well as the expansion of urban centres leading to more demand for efficient and humane poultry production systems [5,6]. As of 2022, the poultry sector is estimated to have a total number of around 82.5 million poultry, with the majority comprising ‘exotic’ chicken at 50.7%, followed by Indigenous Chicken at 36.9%, Crossbreeds at 3.9%, Guinea fowl at 5.6%, Ducks at 1.3%, with the remaining comprising a few other species such as goose, pigeon, amongst others [7,8]. Chicken makes up 90% of the domesticated poultry reared in Ghana [7]. The estimated average annual consumption of poultry meat in Ghana per head of the population increased from 5.5 kg in 2016 to 13.7 kg in 2021 [3,6], indicating a substantial demand and expected growth in line with the growing population. It is essential for the development of market and framing policy decisions to understand the perception of the consumer with respect to poultry meat. Recent studies indicate a rise in demand for beef products globally, emphasizing convenience, variety, safety, and health benefits [9,10]. Due to this shift in consumer preferences, there is now a demand for strict production and labeling standards in Ghana. For example, organic products [11] and beef food safety assurance labels have been implemented in Kumasi Metropolis and Sunyani Municipality [12].

This shift in purchasing behaviour presents a significant opportunity to improve welfare standards in chicken farming [13] because of the current focus on sustainable living and ethical procurement. The Ghanaian government has started the Poultry Intensification Scheme to address the country’s over-reliance on imported processed chicken. The scheme is part of the Food Systems Resilience Programme and the Ministry of Food and Agriculture (MoFA-FSRP) [14,15]. The World Bank funds this program, which is implemented in stages. Each recipient receives input credits that include about 160,000-day-old chicks, 4.5 kg of feed for each bird, and the vaccinations that a flock needs. Furthermore, to emphasise the importance of integrating animal welfare issues in the country’s agricultural development strategy, farmers in the chicken business are trained in the best modern methods and climate-smart technologies.

While Ghana’s poultry industry continues to evolve, it is relevant to holistically consider and address the challenges, including the lack of egg labels in the current system. The current ecosystem of commercial poultry farming, particularly for laying hens, includes housing laying hens in two broad systems: cages and floor housing called the “Deep Litter System.” The battery cage system has been found to be associated with welfare issues. The cages prevent hens from performing the bulk of their natural behaviour, including nesting, perching, dustbathing, scratching, foraging, exercising, running, jumping, flying, stretching, and wing flapping [16]. As a result, they can experience frustration and emotional distress, which may be exhibited by stereotypic back-and-forth pacing behaviour [17]. Unlike the cage system, the deep litter, which is a cage-free housing system, allows for greater locomotive function and the ability to perform innate behaviours such as nesting, flying, and jumping [16,17]. In Ghana, the demand for chicken meat and eggs from these two commercial systems is rising due to rapid urbanisation and accelerating economic growth, and there is no disparity in the pricing of meat and eggs from these systems or from the indigenous chicken; hence, perceptions of poultry products from the perspective of welfare need to be understood. The first research in Ghana to consider the views of Ghanaians regarding farmed poultry welfare and the associated egg product is presented in this study. It is important to understand what citizens and consumers value in an informed way, and this will impact the manner in which people shop for farmed poultry, such as eggs, and how much they are willing to pay in Ghana.

The key research questions for this study were as follows:

1. What are Ghanaians’ perceptions of animal welfare, and do they differ significantly between regions?
2. What demographic factors associate with and predict a positive perception toward animal welfare?
3. What are Ghanaians’ perceptions of higher welfare egg-labelling?

## 2. Methodology

### 2.1. Study Sample

This study used a cross-sectional survey design to assess the perception of Ghanaians on the welfare of farmed animals, especially hens. Ghanaians in the chosen major cities of Tamale, Kumasi, and Accra who were at least 17 years old made up the study’s target demographic. Each of the three cities functions as the regional capital. Accra serves as the administrative centre and commercial hub for the entire nation, serving the Greater Accra Region. Kumasi is the main city of the Ashanti region, the second most populous urban centre in the nation. The third largest city is Tamale, the capital of the northern region of the country [18]. These cities have been chosen for their significance to the poultry sector, as the public perception in these large areas will be important for consideration by the poultry sector. Participants were also considered based on the geographical diversity of the cities, with different cities having variations in the level of economic growth, climate conditions, and the size of the populations to ensure the sample was representative of the Ghanaian cities. Nevertheless, to ensure the representation of those in the study, the sample size was measured using the formula by Cochran to estimate the proportions [19]. The initial calculation, assuming a 95% confidence level, a margin of error of 3%, and an expected proportion of 50% (which maximizes the sample size), yielded a sample size of 1068 (n = Z^2^ × p × (1 − p)/e^2^, where: n is the sample size; Z is the z-score (1.96 for a 95% confidence level); p is the estimated proportion (0.50); e is the margin of error (0.03); n = (1.96^2^ × 0.50 × (1 − 0.50))/(0.03^2^) = (3.8416 × 0.25)/0.0009 = 1068).

### 2.2. Data Collection

#### 2.2.1. Questionnaire Design

In this study, a structured questionnaire adapted from a previous study [2,20,21] was used to assess Ghanaians’ perception of farmed poultry welfare in Ghana, with a particular emphasis on laying hen and egg production. It began with simple sociodemographic questions which focused on participants’ age, gender, religion, ethnicity (classified as Ga, Akan, Northerner, Ewe/Fante, and other based on the major ethnic groupings in Ghana), highest educational background, number of people living in a household, pet ownership, and consumption of chicken and other farm products, including beef, goat, egg, and milk. The first section included a total of nine Likert-scale statements, which provided insight into the perception and predictors of perception toward farmed animal welfare, and the second section started with a preamble and included four closed questions on Ghanaian consumer perception toward higher welfare labelling eggs, all of which were based on validated questions from previous literature [2,21].

Before administering the questionnaire, a pilot test was conducted on 11 individuals outside of the study sites to determine the clarity and usefulness of the questionnaire instrument. A Cronbach’s alpha value of 0.70 was obtained from the pilot study, indicating acceptable internal consistency of the elements of the questionnaire [22]. Based on the pilot study results, minor adjustments were made to improve the clarity and reliability of the survey questions; for instance, specifying the study location in cities of interest.

Participants were given scores based on their responses to the questions [23]. The questions were all Likert-type questions with five possible responses, ranging from strongly disagree to strongly agree. A positive direction was used in the scoring, with a positive maximum response gaining a score of 5 per question and a negative minimum response gaining a score of 1 per question. The maximum score was 45, while the lowest was 9. The participants were classified as having a poor perception toward farmed animal welfare if they had a low composite score of 9–34 (≤75.0%), and participants who had higher composite scores of 35–45 (>75.0%) were classified as having a positive perception toward farmed animal welfare [23]. A ‘positive’ level equates to a positive perception, and a ‘negative’ level equates to poor perception. The full questionnaire is included as Supplementary Material.

#### 2.2.2. Procedure

Between the dates of July 2024 and August 2024, enumerators were deployed to various public spaces in the selected cities, including universities, work offices, and market squares, to facilitate a broad reach among potential respondents. The survey was conducted in-person and data were collected using tablets or mobile devices. This method was chosen to improve accessibility and ensure that respondents from various educational backgrounds could easily participate [20].

### 2.3. Data Analysis

The data collected was exported, cleaned, and coded using Microsoft Excel 2021 version 2108. The statistical analysis was performed using GraphPad Prism version 8.0 (GraphPad Software, San Diego, CA, USA, www.graphpad.com) and the Statistical Package for Social Sciences (SPSS) version 26.0 (Chicago, IL, USA). Categorical variables were presented using percentages and frequencies. A bar chart was used to summarize the various points of view on the welfare of animals raised for food. To determine correlations between categorical variables, the Chi-square test or Fisher’s exact test was also employed. Both univariate and multivariate logistic regression prediction models were used to identify factors associated with positive perceptions of farmed animal welfare. Crude odds ratios (cOR) were obtained from univariate models, and adjusted odds ratios (aOR) were obtained from multivariate models after controlling for potential confounders including age, gender, and other covariates. *p*-values less than 0.05 and a 95% confidence interval were considered statistically significant.

## 3. Results

### 3.1. Demographics and Baseline Characteristics

A total of 1275 Ghanaians participated in the survey, with 34.1% from Accra, 33.3% from Kumasi, and 32.5% from Tamale. Half (50.3%) were between 20 and 25 years of age, with the next highest age group being 31–78 years (19.2%). More than half of the participants were males (53.5%), and 46.5% were females. Moreover, nearly three-quarters were Christians (72.8%), and roughly one-quarter belonged to the Islamic religion (25.5%). Approximately half of the participants achieved a tertiary education (50.5%), and 39.4% achieved a Senior High School educational level. The average household number of participants was 6.6 ± 4.52, and most had 1–5 in the household (49.7%) or 6–10 in the household (39.0%). In addition, 34.0% of participants indicated that they were the people who usually purchased food in their household. Also, 34.2% of participants own a pet or domestic animal such as a dog (48.7%) and a cat (39.1%). Additionally, a majority of participants use chickens or other farmed animals apart from fish in their food daily (46.1%) and 3–5 times a week (29.2%) (Table 1).

### 3.2. Perception Toward Farmed Animal Welfare

The results in Table 2 indicate that the majority of participants “agreed” with the following: *I consider the well-being of farmed animals when they make decisions about purchasing meat, eggs, and milk* (26.9% in the overall sample), *hens should live lives free from pain* (38.1% in the overall sample), *farmers and food companies put their own profits ahead of treating farmed animals well* (36.0% in the overall sample), and *the average Ghanaian thinks that farmed animal welfare is important* (30.7% in the overall sample). These responses varied significantly across the study participants from the different study cities (*p* < 0.05).

In addition, the majority ‘strongly agreed’ with the following: *the government should take an active role in promoting farmed animal welfare* (53.7% in the overall sample). This response was significantly different across the study cities (*p* = 0.0240), whereas participants across the different study cities strongly agreed to a similar extent that *food companies requiring farmers to treat their animals better are doing the right thing* (43.0% in the overall sample). Moreover, there were significant differences in the responses across the study cities regarding the extent of strongly disagreeing that *low meat prices are more important than the well-being of farmed animals* (40.6% in the overall sample; *p* < 0.0001), but expressed neutral views regarding whether *housing chickens in cages is inhumane* (28.1% in the overall sample; *p* < 0.0001) (Table 2).

**Figure 1:**
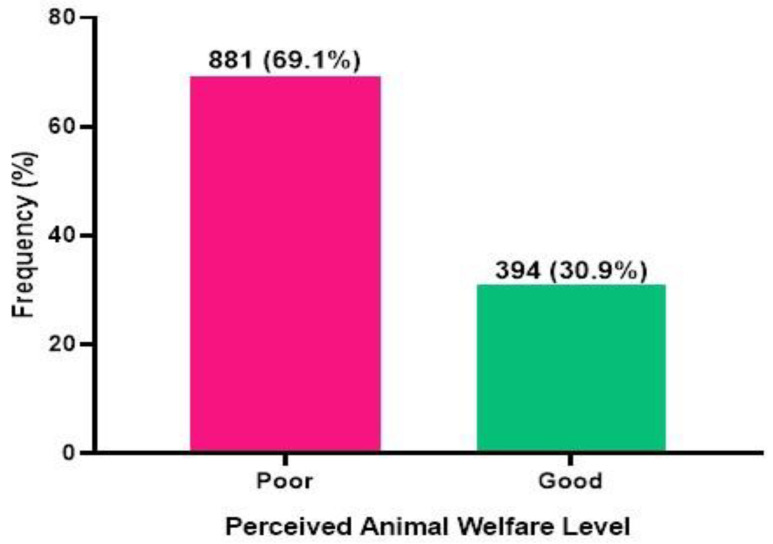
Composite scores regarding perception toward farmed animal welfare.

Acceding to a cut-off of 75.0% as the higher end [23], this study further observed that 30.9% had a positive perception toward farmed animal welfare, whilst 69.1% had a poor perception toward farmed animal welfare (Figure 1).

### 3.3. Factors Associated with Perception Toward Farmed Animal Welfare

In a univariate logistic regression prediction model, compared to Ga, ethnic groups such as Northerners (cOR: 2.03, 95% CI: (1.19–3.46); *p* = 0.009), Ewe/Fante (cOR: 2.02, 95% CI: (1.12–3.63); *p* = 0.019), or other ethnic groups including Bono, Sefwi, Nzema, and Akyem (cOR: 2.28, 95% CI: (1.25–4.16); *p* = 0.007) were significantly associated with approximately twofold increased odds of a positive perception toward farmed animal welfare. Moreover, owning a pet or domestic animal (cOR: 1.39, 95% CI: (1.08–1.77); *p* = 0.010) was significantly associated with an increased likelihood of a positive perception toward farmed animal welfare.

In a multivariate logistics regression prediction model, after adjusting for age, gender, and other confounders, ethnic groups such as Northerners (aOR: 2.11, 95% CI: (1.23–3.63); *p* = 0.007), Ewe/Fante (aOR: 1.98, 95% CI: (1.10–3.57); *p* = 0.023), or other ethnic groups including Bono, Sefwi, Nzema, and Akyem (aOR: 2.37, 95% CI: (1.29–4.36); *p* = 0.005) were independent predictors of increased likelihood of a positive perception toward farmed animal welfare. Similarly, owning a pet or domestic animal (aOR: 1.41, 95% CI: (1.10–1.81); *p* = 0.008) was an independent predictor of increased likelihood of a positive perception toward farmed animal welfare (Table 3).

This study found that, when asked to choose their preferred ad slogan, the highest proportion of participants favoured, “Give chickens the life they deserve. Buy free-range eggs.” (31.9% in the overall sample) and would consider higher welfare egg-labelling when purchasing eggs (68.2% in the overall sample). These responses were similar and significant among participants across the different study cities (*p* < 0.05). Moreover, the majority of participants think that a free-range egg-labelling system would be helpful for consumers (80.3% in the overall sample), but more than half indicated they would not be willing to pay more for cage-free or free-range eggs (52.0% in the overall sample). These responses, however, varied significantly among participants across the different study cities, with 55.8% of respondents from Kumasi being willing to pay as compared to 60.7% of respondents from Accra who were not willing to pay more (*p* < 0.05) (Table 4).

## 4. Discussion

This study describes the perception of young Ghanaians toward farmed poultry welfare, particularly laying hen and egg production systems, and their willingness to support and pay more for welfare-friendly labelled products as consumers. This study has, in particular, established the perception toward farmed poultry welfare, particularly laying hens, among the younger Ghanaians, as 50.3% of the participants were between the ages of 20 and 25 years.

The findings of the study indicate that a high percentage of the participants (69.1%) perceived that the welfare of farmed animals is poorly perceived, while only 30.9% of the participants perceived that the perception of animal welfare is positive. The results highlight a gap in the knowledge of animal welfare issues among the general population. These findings align with the existing literature, which indicates a low awareness or inconsistent views regarding animal welfare support in developing countries, where economic concerns usually outweigh ethical considerations [24]. To realise this opportunity, government interventions are necessary. In the past, various interventions have played important roles in ensuring that the problems in the poultry sector in Ghana are resolved. These include the implementation of an import duty of 35% in 2016 to support and protect local producers and markets [25,26,27].

Notably, opinions about animal welfare varied significantly between cities; participants from Tamale were somewhat more inclined to have a favourable opinion of farmed animal care than participants from Accra and Kumasi. Cultural differences, Tamale residents’ long history with livestock, or different economic situations could all account for these differences between cities. Tamale is the least economically developed of the three cities. A greater proportion of the population engages in livestock rearing than in Kumasi and Accra [7]. According to [24,28], prior research indicates that less urbanised areas worldwide are likely to have greater direct exposure and contact with animals, which may affect opinions of their welfare.

The results also indicate that 83.8% of the participants agree that the government should be actively involved in the promotion of the welfare of farmed animals, and there is a need for increased welfare in the cities, as presented in the study. This finding suggests that government-led initiatives may gain public support, especially in urban towns, where there is more access to media and public information campaigns [29,30]. The high levels of support for government action on the control of animal welfare are simultaneous with global tendencies in which consumer demands have often led to the imposition of more stringent animal welfare legislation [31]. This strong support for government action may also indicate that citizens tend to place the responsibility for welfare improvements entirely on institutions, possibly reflecting concerns about direct personal involvement, particularly in economic terms. This interpretation gains further relevance in light of the subsequent finding that a majority of respondents were unwilling to accept a price increase for cage-free or free-range eggs.

Additionally, the study indicates that most of the respondents believe that food companies requiring farmers to treat animals better are doing the right thing. This indicates that consumers support the cause of responsible corporate treatment of animals, which could be harnessed as a distinguishing aspect of the products themselves. However, more than half of the population (60.7%) are unwilling to pay a premium for cage-free and free-range eggs, which indicates a disparity between the self-proclaimed sentiments and the reality of buying behavior as practice [32]. This correlates with previous findings that people generally support the cause of animal welfare, yet the concern for money easily overrides any moral principles while selecting products for purchase [32]. Nevertheless, this price flexibility could be influenced by more credible labeling, such as certification, for instance, which was found to increase the attitude toward animal welfare as well as the willingness to pay [33,34].

Another notable finding is the strong interest in a proper egg label. A very strong majority (68.2%) would support such an egg label, and an even larger majority (80.3%) would find such an egg label very useful. This reflects an increasing interest in the production process of food in general, as many people all around the world become increasingly conscious about their food behavior and strive for a morally and responsibly produced life [35,36]. Such a demand in a developing market represents many possible implications for price and animal production. Most respondents appear price-sensitive, and thus producers and retailers must take this into consideration in their pricing decisions to not lose any cost-conscious customers. Another factor would be government subsidies and efforts among animal producers, such as the National Cage-Free Farmers Network and Directory, which can group together and share costs regarding certification and labelling, potentially enabling economies of scale and thereby reducing costs per unit and allowing for competitive pricing.

Cost-efficient producers could benefit from their increased profitability by applying strategies that entail premium pricing as well as market differentiation based on price sensitivity and cost considerations [37]. The emphasis should first be on awareness and understanding of the general public on price sensitivity, before producers and retailers will be able to benefit from value alignment and loyalty. A review paper by [37] indicated that CSR activities, such as ethical branding and welfare labelling, have positively added value to brands and resulted in loyalty, acknowledging that firms that promote their ethical stand through ethical branding and welfare labelling tend to have a stronger level of loyalty and added value to their brands. This also highlights that some of the respondents (40.7%) agree that companies that require their farmers to take care of their animals are doing the right thing.

However, one challenge that is evident from this research is with respect to willingness to pay for improved animal welfare products, where 52.0% of the respondents were unwilling to pay a higher amount for cage-free and free-range eggs. This can be related to a similar study carried out in Nairobi, Kenya, where consumers demonstrated a positive attitude toward poultry animal welfare attributes. However, willingness to pay was highly affected by price sensitivity [38]. This indicates how the participants’ opinions on the welfare of farmed animals and their purchase habits are dynamic. Similar research from Ghana indicates that while customers are drawn to the sustainable and high-quality features of poultry products, price plays a significant role in influencing their purchasing choices [39]. While this study highlights the subjects’ difficulties with affordability, it does not indicate that consumers no longer value information about poultry welfare; rather, it highlights the need for a change in approach that must take into consideration both customer knowledge and affordability [40]. Therefore, these findings suggest that affordability must be balanced with customer knowledge for ethically good products to be made accessible. Ref. [41] suggests that the integration of improved farmed animal welfare into quality concepts such as certification schemes and standardized quality assurance systems provides a practical pathway through which improvements are achieved and valued by consumers. This approach could be explored in Ghana through intentional moves towards awareness and policy integration using the Standard Organization and certifications. Furthermore, the findings on ethnicity revealed that Northerner, Ewe/Fante, and other ethnic groups had significantly higher odds of a positive perception toward farmed animal welfare compared to the Ga ethnic group. This may reflect cultural differences in traditional animal husbandry practices across different ethnic groups in Ghana, as communities in the northern regions and certain southern groups have historically maintained closer relationships with livestock rearing [7,42]. These ethnic variations in welfare perceptions underscore the importance of tailoring welfare awareness campaigns to specific cultural contexts within Ghana [43,44].

The limitation of the study is that emphasis was on younger ages, which may not be a full representation of the general public as a whole. However, the recent Ghana population and housing census indicated that the majority of the population is within the age bracket considered [45]. Also, the study did not consider gender influence, and geriatric concern was not included. Additionally, the use of a 75% cut-off to dichotomize perceptions into positive and poor categories is restrictive, as intermediate or moderately positive perceptions may not be fully captured by this binary classification approach.

## 5. Conclusions

This study provides the first assessment of young Ghanaian adults’ perceptions of poultry welfare, egg-labelling, and willingness to pay for higher-welfare eggs across three major cities. The findings reveal a notable gap between welfare attitudes and purchasing behaviour, with price sensitivity emerging as a dominant barrier to the uptake of higher-welfare egg products. Pet ownership and ethnicity emerged as significant predictors of positive welfare perceptions, with Northerners, Ewe/Fante, and other ethnic groups indicating higher odds compared to the Ga group. Regional differences were also observed, with respondents in Tamale demonstrating slightly more positive perceptions than those in Accra and Kumasi, possibly reflecting closer traditional ties to livestock. These findings suggest that producers, retailers, the government, and other stakeholders could work together to establish government subsidies, cost-sharing among producers, certifications, and labelling schemes to provide more affordable, value-aligned, higher-welfare poultry, particularly regarding eggs, to consumers in Ghana. Future research should explore broader age demographics, rural populations, and the potential role of education-based interventions in bridging the gap between welfare attitudes and consumer behaviour in Ghana.

## Figures and Tables

**Table 1:** Absolute (n = 1275) and relative (%) frequencies of demographic information and baseline characteristics of study participants in relation to keeping animals.

Variable	Frequency (n)	Percentage (%)
City		
Accra	435	34.1
Kumasi	425	33.3
Tamale	415	32.5
Age (Years) (μ ± SD)	26.16 ± 9.10	-
Age Group (Years)		
≤19	189	14.8
20–25	641	50.3
26–30	200	15.7
31–78	245	19.2
Gender		
Male	682	53.5
Female	593	46.5
Religion		
Christian	928	72.8
Islam	325	25.5
Traditionalist/Others	22	1.7
Ethnicity		
Ga	100	7.9
Akan	473	37.2
Northerner	398	31.3
Ewe/Fante	167	13.1
Others	135	10.6
Highest Educational Level		
Primary	33	2.6
Junior High School	96	7.5
Senior High School	502	39.4
Tertiary	644	50.5
Average Household Number	6.64 ± 4.52	-
Household Number Category		
1–5	634	49.7
6–10	497	39.0
11–19	103	8.1
≥20	41	3.2
Are you the person who usually purchases food in your household?		
No	842	66.0
Yes	433	34.0
Do you own a pet or domestic animal?		
No	839	65.8
Yes	436	34.2
Which specific pet or domestic animal?		
Dog	207	48.7
Cat	166	39.1
Dog and cat	28	6.6
Domestic animals (sheep, goat, chicken)	24	5.6
How often do you use chickens or other farm animals apart from fish in your foods?		
Never	32	2.5
Once a week	135	10.6
Twice a week	148	11.6
3–5 times a week	372	29.2
Daily	588	46.1

**Table 2:** Absolute (n = 1275) and relative frequencies (%) of responses to animal welfare questions.

Variable	Total (n = 1275)	Accra (n = 435)	Kumasi (n = 425)	Tamale (n = 415)	Test Statistics	*p*-Value
I consider the well-being of farm animals when I make decisions about purchasing meat, eggs, and milk.					46.516	**<0.0001**
Strongly disagree	195 (15.3)	64 (14.7)	82 (19.3)	49 (11.8)		
Disagree	176 (13.8)	73 (16.8)	66 (15.5)	37 (8.9)		
Neutral	259 (20.3)	112 (25.7)	64 (15.1)	83 (20.0)		
Agree	343 (26.9)	111 (25.5)	109 (25.6)	123 (29.6)		
Strongly agree	302 (23.7)	75 (17.2)	104 (24.5)	123 (29.6)		
Low meat prices are more important than the well-being of farm animals.					29.276	**<0.0001**
Strongly disagree	518 (40.6)	155 (35.6)	172 (40.5)	191 (46.0)		
Disagree	347 (27.2)	134 (30.8)	94 (22.1)	119 (28.7)		
Neutral	192 (15.1)	77 (17.7)	62 (14.6)	53 (12.8)		
Agree	129 (10.1)	40 (9.2)	58 (13.6)	31 (7.5)		
Strongly agree	89 (7.0)	29 (6.7)	39 (9.2)	21 (5.1)		
Housing chickens in cages is inhumane.					28.731	**<0.0001**
Strongly disagree	188 (14.7)	51 (11.7)	70 (16.5)	67 (16.1)		
Disagree	255 (20.0)	93 (21.4)	73 (17.2)	89 (21.4)		
Neutral	358 (28.1)	114 (26.2)	104 (24.5)	128 (30.8)		
Agree	291 (22.8)	114 (26.2)	92 (21.6)	85 (20.5)		
Strongly agree	183 (14.4)	51 (11.7)	86 (20.2)	46 (11.1)		
Hens should live lives free from pain.					19.78	**0.0110**
Strongly disagree	68 (5.3)	22 (5.1)	25 (5.9)	21 (5.1)		
Disagree	104 (8.2)	40 (9.2)	37 (8.7)	27 (6.5)		
Neutral	222 (17.4)	89 (20.5)	52 (12.2)	81 (19.5)		
Agree	486 (38.1)	172 (39.5)	159 (37.4)	155 (37.3)		
Strongly agree	395 (31.0)	112 (25.7)	152 (35.8)	131 (31.6)		
Farm animals are less affected by pain and discomfort than humans.					38.341	**<0.0001**
Strongly disagree	284 (22.3)	76 (17.5)	114 (26.8)	94 (22.7)		
Disagree	321 (25.2)	127 (29.2)	81 (19.1)	113 (27.2)		
Neutral	285 (22.4)	95 (21.8)	84 (19.8)	106 (25.5)		
Agree	248 (19.5)	102 (23.4)	85 (20.0)	61 (14.7)		
Strongly agree	137 (10.7)	35 (8.0)	61 (14.4)	41 (9.9)		
Food companies that require farmers to treat their animals better are doing the right thing.					9.177	0.3280
Strongly disagree	63 (4.9)	19 (4.4)	19 (4.5)	25 (6.0)		
Disagree	75 (5.9)	24 (5.5)	26 (6.1)	25 (6.0)		
Neutral	152 (11.9)	66 (15.2)	43 (10.1)	43 (10.4)		
Agree	437 (34.3)	149 (34.3)	153 (36.0)	135 (32.5)		
Strongly agree	548 (43.0)	177 (40.7)	184 (43.3)	187 (45.1)		
The government should take an active role in promoting farm animal welfare.					17.671	**0.0240**
Strongly disagree	51 (4.0)	20 (4.6)	10 (2.4)	21 (5.1)		
Disagree	49 (3.8)	16 (3.7)	13 (3.1)	20 (4.8)		
Neutral	106 (8.3)	33 (7.6)	27 (6.4)	46 (11.1)		
Agree	384 (30.1)	123 (28.3)	130 (30.6)	131 (31.6)		
Strongly agree	685 (53.7)	243 (55.9)	245 (57.6)	197 (47.5)		
Farmers and food companies put their own profits ahead of treating farm animals well.					28.863	**<0.0001**
Strongly disagree	89 (7.0)	19 (4.4)	28 (6.6)	42 (10.1)		
Disagree	131 (10.3)	38 (8.7)	39 (9.2)	54 (13.0)		
Neutral	266 (20.9)	89 (20.5)	76 (17.9)	101 (24.3)		
Agree	459 (36.0)	162 (37.2)	164 (38.6)	133 (32.0)		
Strongly agree	330 (25.9)	127 (29.2)	118 (27.8)	85 (20.5)		
The average Ghanaian thinks that farm animal welfare is important					32.756	**<0.0001**
Strongly disagree	132 (10.4)	33 (7.6)	56 (13.2)	43 (10.4)		
Disagree	216 (16.9)	64 (14.7)	60 (14.1)	92 (22.2)		
Neutral	263 (20.6)	85 (19.5)	77 (18.1)	101 (24.3)		
Agree	392 (30.7)	151 (34.7)	129 (30.4)	112 (27.0)		
Strongly agree	272 (21.3)	102 (23.4)	103 (24.2)	67 (16.1)		

Note: Data are presented as frequencies (and percentages), *p*-values are computed by Chi-square test, *p*-values < 0.05 and bold denotes statistical significance.

**Table 3:** Predictors of perception toward farmed animal welfare.

Variable	Poor (n = 881)	Positive (n = 394)	Test Statistics	cOR (95% CI)	*p*-Value	aOR (95% CI)	*p*-Value
City			2.446				
Accra	309 (35.1)	126 (32.0)		1.00	-	-	-
Kumasi	297 (33.7)	128 (32.5)		1.06 (0.79–1.42)	0.711	-	-
Tamale	275 (31.2)	140 (35.5)		1.25 (0.93–1.67)	0.134	-	-
Age Group (Years)			3.579				
14–19	123 (14.0)	66 (16.8)		1.00	-	1.00	-
20–25	448 (50.9)	193 (49.0)		0.80 (0.57–1.13)	0.210	0.80 (0.56–1.13)	0.197
26–30	146 (16.6)	54 (13.7)		0.69 (0.45–1.06)	0.092	0.74 (0.47–1.14)	0.172
31–78	164 (18.6)	81 (20.6)		0.92 (0.62–1.37)	0.685	0.98 (0.65–1.47)	0.911
Gender			1.131				
Male	480 (54.5)	202 (51.3)		1.00	-	1.00	-
Female	401 (45.5)	192 (48.7)		1.14 (0.90–1.44)	0.288	1.15 (0.90–1.47)	0.263
Religion			2.204				
Christian	652 (74.0)	276 (70.1)		1.00	-	-	-
Islam	214 (24.3)	111 (28.2)		1.23 (0.94–1.60)	0.139	-	-
Traditionalist/Others	15 (1.7)	7 (1.8)		1.10 (0.45–2.73)	0.833	-	-
Ethnicity			10.598				
Ga	80 (9.1)	20 (5.1)		1.00	-	1.00	-
Akan	338 (38.5)	135 (34.3)		1.60 (0.94–2.71)	0.083	1.58 (0.93–2.69)	0.093
Northerner	264 (30.0)	134 (34.0)		2.03 (1.19–3.46)	0.009	2.11 (1.23–3.62)	**0.007**
Ewe/Fante	111 (12.6)	56 (14.2)		2.02 (1.12–3.63)	0.019	1.98 (1.10–3.57)	**0.023**
Others	86 (9.8)	49 (12.4)		2.28 (1.25–4.16)	0.007	2.37 (1.29–4.35)	**0.005**
Highest Educational Level			3.99				
Primary	25 (2.8)	8 (2.0)		1.00	-	-	-
Junior High School	73 (8.3)	23 (5.8)		0.99 (0.39–2.48)	0.974	-	-
Senior High School	350 (39.7)	152 (38.6)		1.36 (0.60–3.08)	0.465	-	-
Tertiary	433 (49.1)	211 (53.6)		1.52 (0.68–3.43)	0.311	-	-
Household Number Category			2.591				
1–5	443 (50.3)	191 (48.5)		1.00	-	-	-
6–10	346 (39.3)	151 (38.3)		1.01 (0.78–1.31)	0.926	-	-
11–19	64 (7.3)	39 (9.9)		1.41 (0.92–2.18)	0.117	-	-
≥20	28 (3.2)	13 (3.3)		1.08 (0.55–2.12)	0.831	-	-
Are you the person who usually purchases food in your household?			2.686				
No	569 (64.6)	273 (69.3)		1.00	-	-	-
Yes	312 (35.4)	121 (30.7)		0.81 (0.63–1.04)	0.102	-	-
Do you own a pet or domestic animal?			6.705				
No	600 (68.1)	239 (60.7)		1.00	-	1.00	-
Yes	281 (31.9)	155 (39.3)		1.39 (1.08–1.77)	0.010	1.41 (1.10–1.81)	**0.008**
What specific pet or domestic animal?			2.49				
Dog	127 (46.5)	80 (52.6)		1.00	-	-	-
Cat	110 (40.3)	56 (36.8)		0.81 (0.53–1.24)	0.328	-	-
Dog and cat	21 (7.7)	7 (4.6)		0.53 (0.22–1.30)	0.166	-	-
Domestic animals (sheep, goat, chicken)	15 (5.5)	9 (5.9)		0.95 (0.40–2.28)	0.913	-	-
How often do you use chickens or other farm animals apart from fish in your foods?			1.533				
Never	24 (2.7)	8 (2.0)		1.00	-	-	-
Once a week	90 (10.2)	45 (11.4)		1.50 (0.62–3.60)	0.822	-	-
Twice a week	100 (11.4)	48 (12.2)		1.44 (0.60–3.44)	0.673	-	-
3–5 times a week	254 (28.8)	118 (29.9)		1.39 (0.61–3.19)	0.615	-	-
Daily	413 (46.9)	175 (44.4)		1.27 (0.56–2.89)	0.329	-	-

Note: Model is adjusted by age and gender, *p*-values are computed by logistics regression prediction model, *p*-values < 0.05 and bold denotes statistical significance.

**Table 4:** Responses to questions on higher welfare egg-labelling.

Variable	Accra (n = 435)	Kumasi (n = 425)	Tamale (n = 415)	Test Statistics	*p*-Value
Imagine you saw an ad for free-range eggs. Which of these options most appeals to you?				10.128	0.1190
Be part of the change and buy free range!	112 (25.7)	87 (20.5)	106 (25.5)		
Give chickens the life they deserve. Buy free-range eggs.	119 (27.4)	145 (34.1)	143 (34.5)		
Let’s make life without discomfort the norm. Buy free-range eggs.	82 (18.9)	80 (18.8)	66 (15.9)		
Say no to chicken suffering. Buy free-range instead.	122 (28.0)	113 (26.6)	100 (24.1)		
Imagine that all supermarkets in your neighbourhood instituted a new egg-labelling system where eggs from chickens raised without cages were labelled “cage-free”, eggs from chickens raised outside were labelled “free range”, and eggs from factory farms remained unlabelled I would consider this egg-labelling system when purchasing eggs.				4.227	0.1210
No	148 (34.0)	119 (28.0)	138 (33.3)		
Yes	287 (66.0)	306 (72.0)	277 (66.7)		
I would be willing to pay more for cage-free or free-range eggs.				23.648	**<0.0001**
No	264 (60.7)	188 (44.2)	211 (50.8)		
Yes	171 (39.3)	237 (55.8)	204 (49.2)		
I think this egg-labelling system would be helpful for consumers.				10.786	**0.0050**
No	106 (24.4)	66 (15.5)	79 (19.0)		
Yes	329 (75.6)	359 (84.5)	336 (81.0)		

Note: Data are presented as frequencies (and percentages), *p*-values are computed by Chi-square test, *p*-values < 0.05 and bold denotes statistical significance.

## Data Availability

Data used in this study are available on request from the corresponding author.

## Supplementary Materials

The following supporting information can be downloaded at: https://www.mdpi.com/article/10.3390/ani16071120/s1. The full questionnaire is included as supplementary.

## Abbreviations

The following abbreviations are used in this manuscript:

MoFA	Ministry of Food and Agriculture
FSRP	Food Systems Resilience Programme
SPSS	Statistical Package for Social Sciences
CSR	Corporate Social Responsibility
