# Supplementary material for: Insights into Public Perception Towards Poultry Welfare, Egg Labelling, and Willingness to Pay Among Young Adults in Ghana
Source: Animals (Basel). 2026 Apr 7;16(7):1120. doi: 10.3390/ani16071120 (PMC13072303; doi:10.3390/ani16071120)
Supplement: Supplementary file 1 [file animals-16-01120-s001.zip › animals-4140544-supplementary.pdf]

Public Opinion on Living Conditions of Laying Hens

This survey is to assess Ghanaians' overall attitudes and opinions towards laying hens and other farmed chicken. It specifically examines the living conditions of hens and their treatment and welfare during production.

Please note that the survey is for research purposes only; you should have signed a consent waiver form on the previous page detailing the purpose and aims of this study. Again, kindly note that the information recorded will not be used for any other purpose.

* Enumerator ID

* Assigned ID

* **City**

○ Accra ○ Kumasi ○ Tamale

* **Age**

* **Gender**

○ Male ○ Female

* **Religion**

☐ Christian ☐ Islam ☐ Traditionalist

☐ Other (Please specify)

* **Ethnicity**

This survey was created on Zoho Survey, an online survey tool. Create unlimited surveys for free on www.zoho.com/survey

* Highest level of school you have completed?

- ◯ None
- ◯ Primary
- ◯ Junior High School
- ◯ Senior High/Technical Vocational School
- ◯ Tertiary

* How many people, including yourself, live in your household?

* Are you the person who usually purchases food in your household?

- ◯ No
- ◯ Yes

* Do you own a pet?

- ◯ No
- ◯ Yes

If own a pet, specify

* How many times per week do you eat chicken, beef, goat, eggs, milk or any other meat or animal product (excluding fish?)

- ◯ Daily
- ◯ 3-5 times a week
- ◯ Twice a week
- ◯ Once a week
- ◯ Never

Please read the following statements (questions 11-19) carefully.

Indicate the level at which you **agree or disagree** with each statement. **Choose 1 if you Strongly Disagree, 2 if you Disagree, 3 if you Neither Agree or Disagree, 4 if you Agree, 5 if you Strongly Agree.**

This survey was created on Zoho Survey, an online survey tool. Create unlimited surveys for free on www.zoho.com/survey

* I consider the well-being of farm animals when I make decisions about purchasing meat, eggs, and milk

Strongly disagree | Strongly Agree

1 2 3 4 5

* Low meat prices are more important than the well-being of farm animals

Strongly Disagree | Strongly Agree

1 2 3 4 5

* Housing chickens in cages negatively affects their wellbeing

Strongly Disagree | Strongly Agree

1 2 3 4 5

* Hens should live lives free from pain

Strongly Disagree | Strongly Agree

1 2 3 4 5

* Farm animals are less affected by pain and discomfort than humans are

Strongly Disagree | Strongly Agree

1 2 3 4 5

* Food companies that require farmers to treat their animals better are doing the right thing

Strongly Disagree | Strongly Agree

1 2 3 4 5

This survey was created on Zoho Survey, an online survey tool. Create unlimited surveys for free on www.zoho.com/survey

* **The government should take an active role in promoting farm animal welfare**

Strongly Disagree | Strongly Agree

1 2 3 4 5

* **Farmers and food companies put their own profits ahead of treating farm animals well**

Strongly Disagree | Strongly Agree

1 2 3 4 5

* **The average Ghanaian thinks that the well-being of farm animals is important**

Strongly Disagree | Strongly Agree

1 2 3 4 5

* **Imagine you saw an ad for free-range eggs. Which of these options most appeals to you**

- Give chickens the life they deserve. Buy free-range eggs.
- Let's make life without discomfort the norm. Buy free range eggs.
- Be part of the change and buy free range!
- Say no to chicken suffering. Buy free range instead

Read carefully the following preamble and use it to attempt the following questions

Imagine that all supermarkets in your neighbourhood instituted a new egg-labelling system where eggs from chickens raised without cages were labelled "cage-free", eggs from chickens raised outside were labelled "free range", and eggs from factory farms remained unlabelled.

* **I would consider this egg-labeling system when purchasing eggs**

- Yes
- No

This survey was created on Zoho Survey, an online survey tool. Create unlimited surveys for free on www.zoho.com/survey

* **I would be willing to pay more for *cage-free* or *free range* eggs**

- ◯ Yes
- ◯ No

* **I think this egg-labelling system would be helpful for consumers**

- ◯ Yes
- ◯ No

This survey was created on Zoho Survey, an online survey tool. Create unlimited surveys for free on www.zoho.com/survey
